# Short-Term Benefits of Robotic Assisted Total Knee Arthroplasty Over Computer Navigated Total Knee Arthroplasty Are Not Sustained With No Difference in Postoperative Patient-Reported Outcome Measures

**DOI:** 10.1016/j.artd.2021.11.014

**Published:** 2022-01-12

**Authors:** Gavin Clark, Richard Steer, Bethany Tippett, David Wood

**Affiliations:** a St John of God Hospital, Subiaco, Western Australia, Australia; b Perth Hip and Knee Clinic, Subiaco, Western Australia, Australia; c University of Western Australia, Medical School, Crawley, Western Australia, Australia; d Department of Orthopaedic Surgery, Gold Coast University Hospital, Southport, Queensland, Australia; e University of Queensland, School of Medicine, St Lucia, Queensland, Australia

**Keywords:** Total knee arthroplasty, Computer navigation, Robotic arm assisted, Clinical outcomes, Length of stay

## Abstract

**Background:**

The purpose of this study was to compare early clinical and patient-reported outcomes between robotic assisted (RA) and computer navigation (CN) total knee arthroplasty (TKA).

**Methods:**

One hundred and fifty patients were enrolled in this prospective, single-surgeon, cohort study, with 75 patients each receiving CN-TKA or RA-TKA in a consecutive series. There were no differences in patient age (*P* = .34) or body mass index (*P* = .09), but a higher proportion of males underwent RA-TKA (*P* = .03). We recorded hospital knee pain, analgesic usage, length of hospital stay, range of motion, and patient-reported outcome measures postoperatively for both patient cohorts.

**Results:**

Hospital length of stay was shorter for the RA-TKA patients (*P* < .001). RA-TKA patients showed improved range of motion (*P* < .001) and decreased pain scores (*P* = .006) on day 1. Subsequent days showed no significant differences. Narcotic usage was lower for the RA-TKA group on day 2 postoperatively (*P* = .03) and onwards. Total morphine equivalent dose was also significantly lower for the RA-TKA than for the CN-TKA group (*P* < .001). There was no difference in Forgotten Joint Score (*P* = .24) or Oxford Knee Score (*P* = .51) between groups at 2 years postoperatively.

**Conclusion:**

The use of RA-TKA demonstrated reduced postoperative analgesia usage and length of stay. There were no differences seen between CN-TKA and RA-TKA with respect to clinical outcomes at 2 years after surgery.

## Introduction

Total knee arthroplasty (TKA) is a highly successful procedure for patients with end-stage osteoarthritis. However, not every TKA provides a patient with an ideal knee postoperatively [[1], [2], [3]]. Patients may complain of unresolved pain or suboptimal function [4,5]. Over the past 2 decades, in an effort to address these issues, computer navigation (CN) and robotics have been introduced. Numerous studies have shown that these advanced technologies improve accuracy and precision of component alignment in TKA [6,7]. But in a cost-saving environment, there is a need to determine if there are longer term benefits apart from achieving a desired implant alignment target.

CN systems for TKA were introduced in the 1990s, and adoption of CN has been varied by region over the past 2 decades [8]. Benefits of CN include no additional preoperative imaging or planning, improved accuracy of implant positioning, and reduced revision rate for patients younger than 65 years [8]. These benefits likely explain the increased use in Australia where the proportion of primary total knee devices implanted with CN had increased from 2.4% in 2003 to 33.5% in 2017 [9]. A 10-year follow-up survivorship study reported 98% survivorship with navigation compared with 93% using manual instruments, using revision surgery as an end point [10]. But, while CN has been shown to reduce surgical outliers in limb alignment, to date, strong clinical data showing improved outcomes in early follow-up for all age groups have yet to be presented [11,12].

Robotics was first developed for TKA in 1992, but the uptake of this technology was limited by its complexity [13,14]. In 2012, the Mako robotic arm-assisted (RA) technology (Stryker, Mahwah, NJ) was first approved for partial knee arthroplasty and was subsequently approved for TKA [15,16]. This haptic RA-TKA system relies on preoperative computed tomography imaging to create a patient-specific 3D model of the knee joint to accurately plan bone resection preoperatively [17].

The saw blade attached to the robotic arm moves within a preplanned stereotactic boundary, which decreases iatrogenic soft-tissue damage [18,19]. Similar to CN, RA-TKA studies have reported improved surgical accuracy and precision of bone cuts compared with manual TKA instruments [20]. The RA and CN techniques have both been shown to be superior to manual instruments [20,21] and have several similarities, for example, both systems allow for intraoperative assessment of joint stability, do not require the use of intramedullary rods, and have been shown to reduce outliers in coronal limb alignment [22,23]. The robotic technique also provides additional information on the medial and lateral gaps [24], but it is unclear whether there is a difference in patient outcomes when comparing RA-TKA to CN-TKA.

RA-TKA has been shown to decrease analgesia requirements, length of stay (LOS), and physical therapy requirements immediately after surgery when compared with the use of conventional instrumentation [25]. Decreased pain has been documented up to 6 months after surgery [26]. It is unclear how much of this benefit would be maintained compared with CN-TKA. If the benefit is derived from the haptic boundary of the saw blade diminishing soft-tissue damage, then the differences should remain.

Therefore, the purpose of this study was to determine if the choice of surgical instrument (CN or RA) had influence on early clinical and patient-reported outcomes. We hypothesize that there would be a difference between these groups as this is what had been shown between RA-TKA and conventional instruments. We recorded hospital LOS and patient-reported outcome measures (PROMS) at 3 months, 1 year, and at a minimum of 2 years follow-up. Moreover, to understand the relevance of soft-tissue protection aspect of the RA technique, we collected inpatient pain scores and analgesia usage, which may be considered surrogates for soft-tissue trauma.

## Material and methods

### Patients

One hundred and fifty patients were enrolled in this prospective, single-center, cohort study. The first group of patients received CN-TKA (Nav 3i; Stryker, Kalamazoo, MI) between December 2015 and November 2016, while the second group received RA-TKA (Mako Robotic-Arm Assisted Surgery System; Stryker, Kalamazoo, MI) between December 2016 and August 2017. All procedures were performed by a single surgeon who has a high-volume TKA practice using both CN- and RA-TKA.

Patients were enrolled in this institutional review board-approved study if they were indicated for a unilateral primary total knee replacement, were able to independently complete PROMS, and did not have a history of chronic pain. Patients in each group were sequential cohorts, with the CN-TKA patients being the 75 patients to have TKA by the surgeon immediately before the introduction of RA-TKA. Intraoperatively, all patients received spinal anesthesia with sedation and an adductor canal block with local anesthetic infiltration of joint capsule and skin. Postoperatively, consistent pain management and rehabilitation protocols were applied to all study subjects. All patients received a combination of paracetamol, celecoxib, slow-release oxycodone (5- to 10-mg bd), and fast-release oxycodone (2.5- to 10-mg 2/24 prn). Dosages were titrated to requirements, and alternatives were used if patients did not tolerate the standard regime. There were no changes in postoperative management during the time in which this study was undertaken. Discharge criteria were standardized. Six patients in each group were discharged to a rehabilitation unit, with all others discharged home.

Alignment targets for both groups were within a boundary of 5° varus to 3° valgus coronal alignment using an adjusted mechanical axis principle. This was achieved with preresection balancing using the software and virtual balancing abilities of both systems. No bony recuts were performed in either group.

The surgeon performed a medial parapatellar approach with minimal soft-tissue releases for exposure. The patella was resurfaced in all cases using a conventional cutting guide. All patients in both groups received cemented tibial and patella components with uncemented femoral components (Triathlon CR Knee System; Stryker, Mahwah, NJ).

### Data collection

Demographic information, history, and examination findings were recorded for all cases. In-hospital metrics included blood loss, LOS, range of motion (ROM) in operated leg, patient-reported Visual Analogue Pain Score (scale 0-10), and the use of medications. Medications recorded included families of analgesics including opioids, nonsteroidal anti-inflammatory drugs, antiemetic, and anaesthetics. The observers were unaware that their recording in hospital data was being used to assess patient outcomes, and it was recorded as part of routine nursing practice. Opioid dosages given to the patients were converted to oral morphine-equivalent dosages [27]. At 90-day, 1-year, and 2-year postoperative visits, Oxford Knee Score and Forgotten Joint Score (FJS) were captured. ROM was recorded by the research physiotherapist using a manual goniometer as part of the standard practice at the clinic.

Descriptive statistics were used to describe means and standard deviations for all variables in this study. A sample size calculation was performed using FJS 2 years after surgery as the primary outcome measure. Using existing data from published studies [28] on TKA, the mean was set at 76 (±23), with the clinical difference set at 12 as the minimal clinically important difference. This study required 60 patients in each arm to detect this minimum difference in FJS using a two-tailed, two-sample t-tests with a power of 80% and significance level of 5%. To account for 20% attrition in sample size within the 2-year follow-up period, 150 patients (75 patients in each treatment group) were included in this study. Student t-tests were applied to all parametric variables with an alpha of 0.05. All statistical analyses were conducted with Stata Statistical Software (SE v15; StataCorp. 2017).

## Results

There was no difference between the CN-TKA and RA-TKA patients in patient age (CN-TKA 67 ± 10 vs RA-TKA 65 ± 8 years, *P* = .34), BMI (CN-TKA 31 ± 5 vs RA-TKA 32 ± 6 kg/m^2^, *P* = .09), or operative side (right:left ratio CN-TKA 32:43 vs RA-TKA 32:43; *P* = .99). There was a higher portion of males who underwent RA-TKA (CN-TKA male:female ratio 24:51 vs RA-TKA 37:38, *P* = .03). There was no difference in mean preoperative European Quality of Life in five dimensions score (EQ-5D) (CN-TKA 67 vs RA-TKA 71; *P* = .19), Oxford Knee Score (OKS) (CN-TKA 22 vs RA-TKA 22; *P* = .90), or FJS (CN-TKA 10 vs RA-TKA 14; *P* = .15) between groups. There was no difference in mean preoperative ROM between groups (CN-TKA 115° vs RA-TKA 116°; *P* = .65).

**Figure 1:**
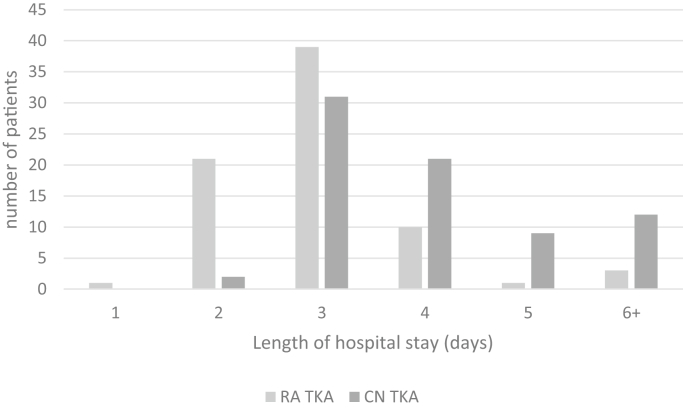
Bar chart showing length of stay for patients having undergone either robotic or navigated TKAs.

**Figure 2:**
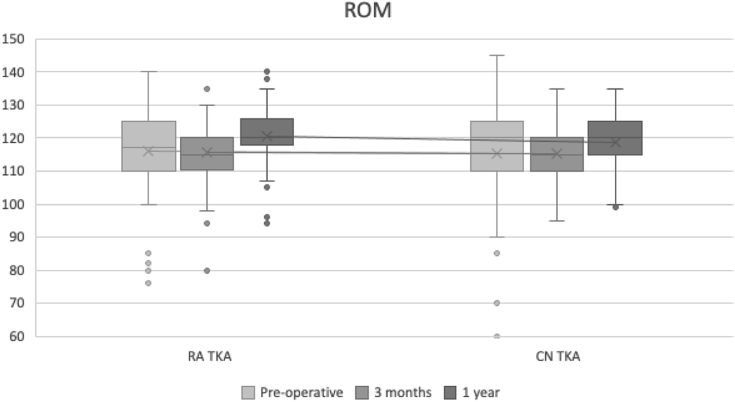
Box and whisker charts at 3 months preoperatively and at 1 year for ROM.

We found a shorter LOS associated with RA-TKA than with CN-TKA (mean 3.1 days vs 4.1 days, *P* < .001; Fig. 1). RA-TKA patients had greater ROM values for day 1 (CN-TKA 84 ± 12 vs RA-TKA 90 ± 7 degrees flexion, *P* < .01; Fig. 2), but this difference did not remain when measured on day 2. RA-TKA patients reported significantly less pain than CN-TKA patients at day 1 after surgery (Visual Analogue Pain Score (0-10) 2.6 compared with 3.4, *P* < .01). Opioid requirement was lower for the RA-TKA group on all days of admission. While the difference did not reach statistical significance on day 1 (*P* = .13), there was a statistically significant decrease in opioid use on days 2-4 (*P* < .01). Total morphine equivalent use was significantly lower for the RA-TKA than for the CN-TKA group (*P* < .01) (Table 1). There were 2 patients (1 in each group) that used little or no opioid because of delirium after their anesthetic.

Time needed to complete the surgical procedure was longer for RA-TKA cases (CN-TKA mean tourniquet time of 70 minutes compared with 86 minutes with RA-TKA; *P* < .01). There were no differences seen in blood loss as measured by change in hemoglobin levels.

**Figure 3:**
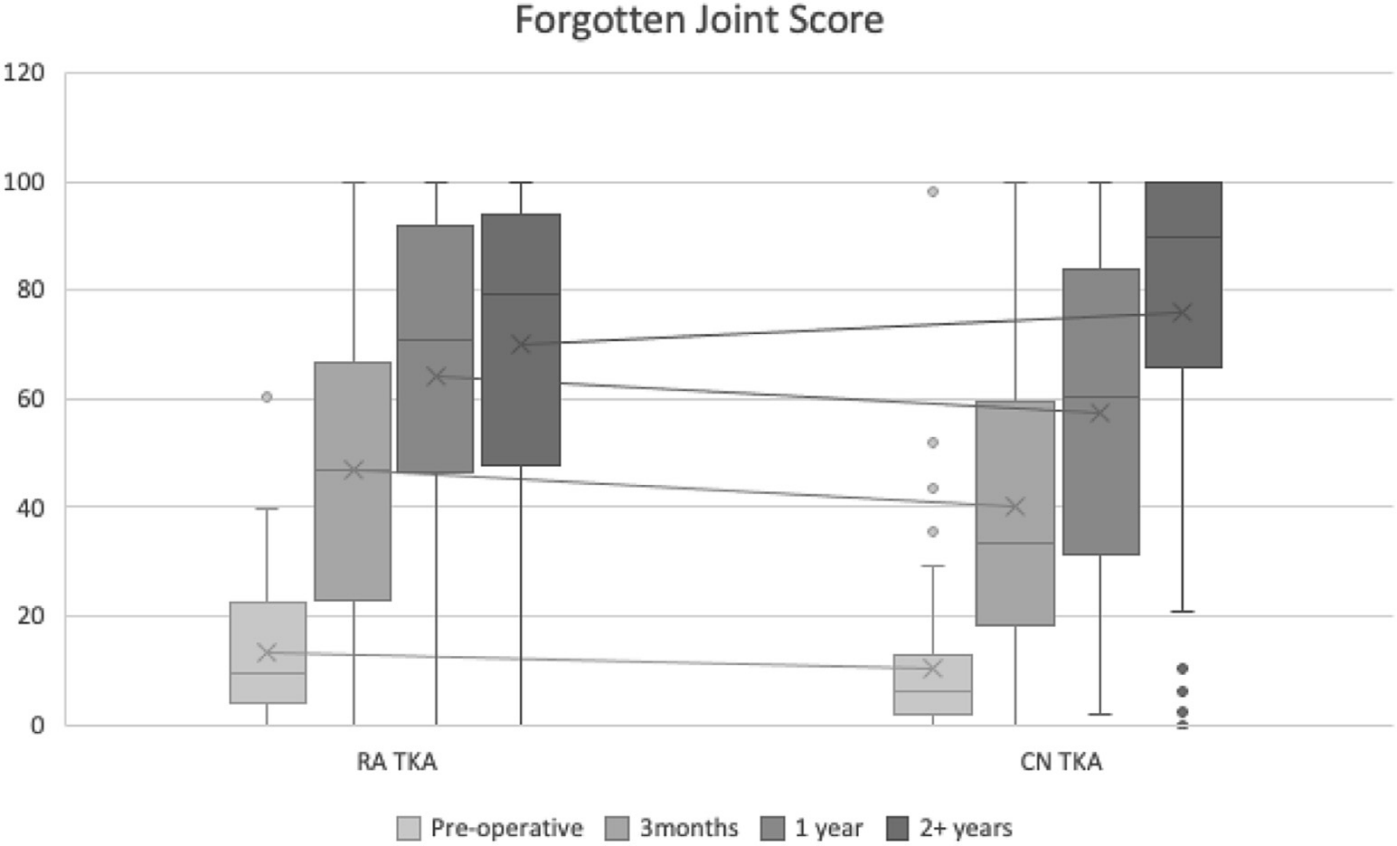
Box and whisker charts at 3 months preoperatively and at 1 year and 2 years for FJS.

**Figure 4:**
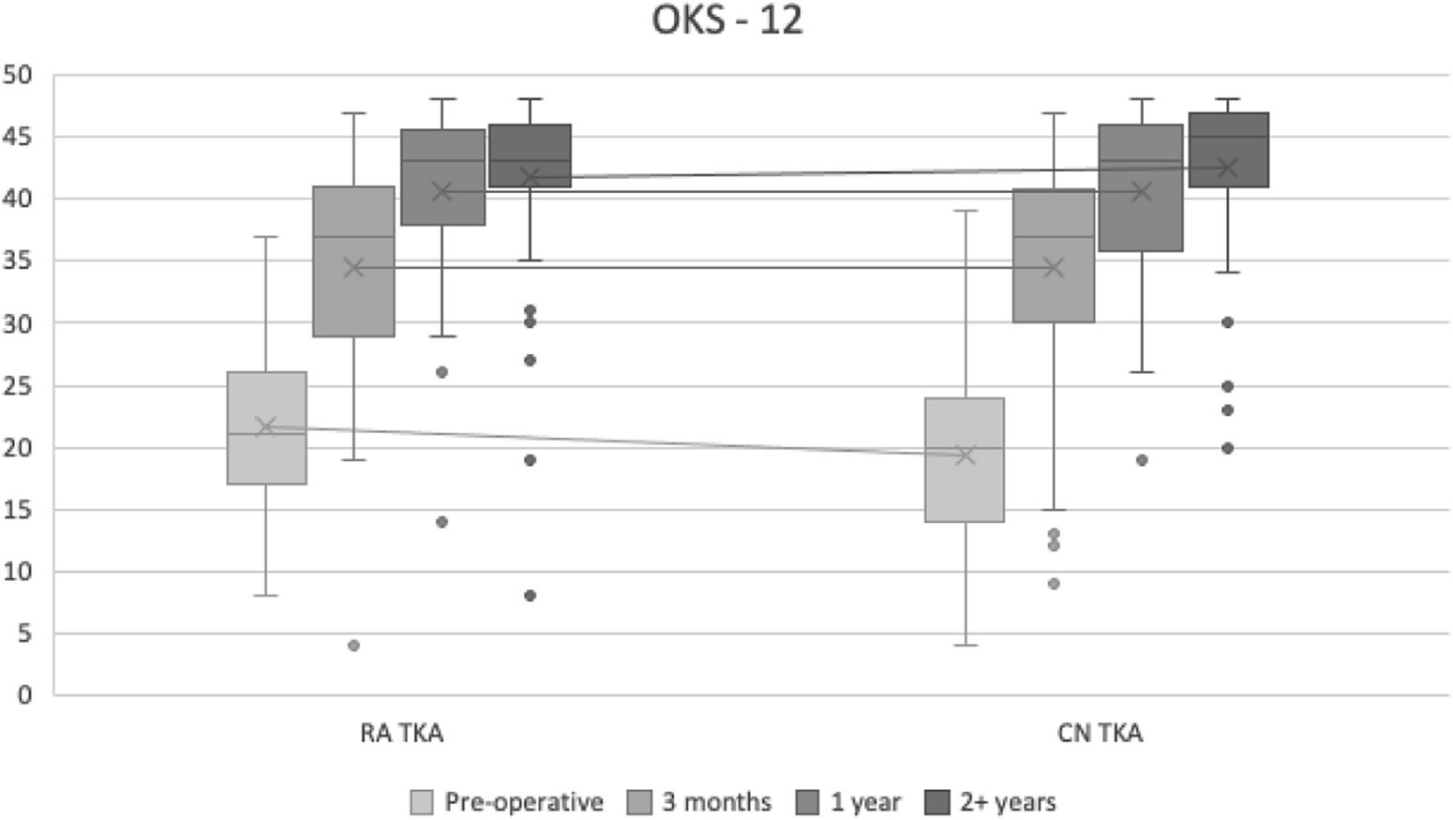
Box and whisker charts at 3 months preoperatively and at 1 year and 2 years for OKS.

At 3 months and 1 year, there was no significant difference in FJS (*P* = .174, *P* = .169) or OKS(*P* = .985, *P* = .975) between groups (Figure 3, Figure 4). One hundred thirty-four of the 150 patients (89%) were assessed at a minimum of 2 years postoperatively (61 CN-TKA and 73 RA-TKA). After 2 years, there was also no significant difference in FJS (*P* = .366; Figure 3, Figure 4) or OKS (*P* = .509; Figure 3, Figure 4) between groups with a longer duration of follow-up in the CN cohort (mean follow-up of 44 months [24-54] compared with 28 months for RA cohort [24-44], *P* < .001). There was no significant difference seen in postoperative ROM at either 3 months (*P* = .912) or 1 year postoperatively (*P* = .512) (Figure 3, Figure 4).

## Discussion

To our knowledge, this is the first study to compare computer-navigated and robotic TKA. We found less opioid use, reduced pain, and shorter LOS postoperatively in patients who underwent RA-TKA compared with those who underwent CN-TKA. We anticipated finding improved short-term patient outcomes with robotics as robotics has been shown to reduce iatrogenic soft-tissue damage and preserve vulnerable structures in the knee capsule [29]. This adds to the evidence for these short-term improvements being due to the haptic controlled saw blade rather than any improvement in accuracy.

The diminished opioid requirements with decreased LOS are likely to be beneficial in view of the increased use of day surgery for TKA and the desire to limit narcotic usage [30]. The resultant decreased LOS is beneficial to overall health-care costs associated with the procedure. Our finding of reduced LOS with robotic TKA has been previously shown in a study comparing robotic TKA to manual TKA [25,31,32]. These differences in pain scores and opioid usage between patient groups may be attributed to 3 technical differences between RA-TKA and CN-TKA. First, RA-TKA provides a stereotactic boundary to constrain the saw blade to make bone cuts. Hampp et al. showed decreased iatrogenic soft-tissue damage with RA-TKA compared with manual TKA in a cadaveric study [19]. A clinical study conducted by Kayani et al. [18] who assessed the soft-tissue damage in 30 consecutive manual TKAs compared with 30 consecutive RA-TKAs found less iatrogenic soft-tissue damage with RA-TKA. This led to improved early straight leg raise, less reported pain, decreased LOS, and a reduction in opioid usage in RA-TKA over conventional instrumentation [18]. Robotic arm TKA reduces the need for tibial subluxation during surgery, which may result in less soft-tissue damage. During RA-TKA, tibial subluxation is only required during component implantation while in CN-TKA, tibial subluxation is also required for tibial resection to protect posterior soft-tissue structures. And finally, RA-TKA used three-dimensional computed tomography for planning and intraoperative balancing, while 1 of the concerns with CN-TKA is inaccurate landmark identification [33]. A hypothetical advantage of three-dimensional planning with RA-TKA is improved tibiofemoral rotational alignment, which may reduce pain after TKA [34,35].

There was an increase in length of procedure between CN-TKA and RA-TKA (16 minutes more with RA-TKA). This would impact operating room efficiency and costs. One of the factors was the increased amount of data collection and research taking part in the RA-TKA cases. Although the learning curve associated with RA surgery has been shown to be short [36], these time differences are still likely to be impacted by the cases being the first 75 undertaken by the surgeon compared with the CN technique, which had been used for over 10 years. The RA-TKA technique has been shown to be time-neutral in numerous studies [37], suggesting further efficiencies can be gained with the RA technique.

While RA-TKA provided benefits in the immediate postoperative period, we did not find a significant difference between CN- and RA-TKA when evaluating postoperative PROMS at any time point between 3 months and 2 years after surgery. Both techniques have been shown to be accurate relative to manual instrumentation and use optic tracking with the ability to verify accuracy intraoperatively [6,20,37]. Given that the alignment philosophy was the same in both groups and the potential differences in accuracy are minimal, it is not surprising the clinical outcomes are similar in the longer term. This would suggest that implant position and limb alignment are likely to have greater impact on longer term outcomes than the instruments used to achieve these goals.

There are several limitations of this study. The procedures were performed sequentially, with CN-TKA being performed on 75 patients before the commencement of RA-TKA at the institution. The RA-TKA group consists of the surgeon’s first 75 cases using this technique and thus include the learning curve within the group. The patients were not blinded to the technique used. We have not included radiological outcomes to compare RA-TKA patients to CN-TKA patients. This study did not examine overall costs of the 2 techniques, and a health economics comparison including the equivalent longer term outcomes remains warranted. Surgeon preference and ease of use considering both physical and mental fatigue would also be relevant to future comparisons.

## Conclusions

This study has demonstrated a significant reduction in postoperative opioid requirement and LOS with the use of RA-TKA when compared with CN-TKA. The short-term differences are not maintained, with no difference in patient-reported outcomes or ROM seen between 3 months and 2 years after surgery. Future prospective randomized controlled trials are warranted to prove this.

## Conflicts of interest

G.W. Clark is in the speakers' bureau of or gave paid presentations for, is a paid consultant for, and receives research support as a principal investigator from Stryker. D. Wood receives royalties from Global Orthopaedics for Absolut hip replacement and receives research support from Zimmer for RCT of onlay vs inset patella.

For full disclosure statements refer to https://doi.org/10.1016/j.artd.2021.11.014.

## Figures and Tables

**Table 1:** Pain and opioid analgesic usage after surgery.

	CN-TKA, mean (range)	RA-TKA, mean (range)	*P* value (Student t-test)
Opioid analgesia day 1^a^	94 (0-243)	80 (0-211)	.13
Opioid analgesia day 2^a^	90 (0-341)	67 (0-218)	**.03**
Opioid analgesia day 3^a^	60 (0-247)	27 (4-137)	**<.01**
Total opioid usage^a^	262 (0-859)	173 (30-511)	**<.01**
Day 0 pain score (NRS 0-10)	1.4 (0-7)	0.9 (0-5)	.06
Day 1 pain score (NRS 0-10)	3.4 (0-7.6)	2.6 (0-6.2)	**<.01**
Day 2 pain score (NRS 0-10)	3.5 (0-8.2)	3.1 (0-7.6)	.23
Day 3 pain score (NRS 0-10)	3.2 (0-7.6)	3.0 (0-6.4)	.78

Bold values represent parameters in which a statistically significant difference was observed (*P* < .05).

a Dosage units are mg in morphine equivalent dosage.

## Supplementary data

Conflict of Interest Statement for Clark

Conflict of Interest Statement for Wood

Conflict of Interest Statement for Tippett

Conflict of Interest Statement for Steer
