# Supplementary material for: Short-Term Benefits of Robotic Assisted Total Knee Arthroplasty Over Computer Navigated Total Knee Arthroplasty Are Not Sustained With No Difference in Postoperative Patient-Reported Outcome Measures
Source: Arthroplast Today. 2022 Jan 12;14:210–215.e0. doi: 10.1016/j.artd.2021.11.014 (PMC9059073; doi:10.1016/j.artd.2021.11.014)
Supplement: Conflict of Interest Statement for Clark [file mmc1.pdf]

INDIVIDUAL CONFLICT OF INTEREST STATEMENT

American Association of Hip and Knee Surgeons

(Adopted from the American Academy of Orthopaedic Surgeons disclosure statement)

The following form **must be filled out completely and submitted by each author (example, 6 authors, 6 forms). All items require a response. If there is no relevant disclosure for a given item, enter "*None.*"**

Manuscript Title: Short Term Benefits of Robotic Assisted Total Knee Arthroplasty over Computer Navigated Total Knee Arthroplasty are Not Sustained with No Difference in Post-Operative Patient Reported Outcome Measures

---

1. Royalties from a company or supplier (The following conflicts were disclosed)

   Nil

2. Speakers bureau/paid presentations for a company or supplier (The following conflicts were disclosed)

   Stryker

3A. Paid employee for a company or supplier (The following conflicts were disclosed)

   Nil

3B. Paid consultant for a company or supplier (The following conflicts were disclosed)

   Stryker

3C. Unpaid consultants for a company or supplier (The following conflicts were disclosed)

   Nil

4. Stock or stock options in a company or supplier (The following conflicts were disclosed)

   Nil

5. Research support from a company or supplier as a Principal Investigator (The following conflicts were disclosed)

   Stryker

6. Other financial or material support from a company or supplier (The following conflicts were disclosed)

   Nil

7. Royalties, financial or material support from publishers (The following conflicts were disclosed)

   Nil

8. Medical/Orthopaedic publications editorial/governing board (The following conflicts were disclosed)

   Nil

9. Board member/committee appointments for a society (The following conflicts were disclosed)

   Nil

Each author must sign AND print or type his/her name, date and submit a separate form

In addition, one BLINDED Conflict of Interest form (no author names used) should be submitted per manuscript with all author disclosures.

Dr Gavin William Clark	Gavin Clark	05/07/2021
Author Name (Print or Type)	Author Signature	Date
